# Combined transplantation of neural stem cells and bone marrow mesenchymal stem cells promotes neuronal cell survival to alleviate brain damage after cardiac arrest *via* microRNA-133b incorporated in extracellular vesicles

**DOI:** 10.18632/aging.103920

**Published:** 2021-01-12

**Authors:** Fang Li, Jie Zhang, Anbao Chen, Rui Liao, Yongchun Duan, Yuwei Xu, Lili Tao

**Affiliations:** 1 Department of Emergency, The Second Affiliated Hospital of Kunming Medical University, Kunming 650000, Yunan Province, P.R. China; 2 The 2nd Department of Hepatobiliary Surgery, The First Affiliated Hospital of Kunming Medical University, Kunming 650000, Yunan Province, P.R. China

**Keywords:** bone marrow mesenchymal stem cells, neural stem cells, extracellular vesicles, microRNA-133b, cardiac arrest

## Abstract

Neural stem cell (NSC) transplantation has prevailed as a promising protective strategy for cardiac arrest (CA)-induced brain damage. Surprisingly, the poor survival of neuronal cells in severe hypoxic condition restricts the utilization of this cell-based therapy. Extracellular vesicles (EVs) transfer microRNAs (miRNAs) between cells are validated as the mode for the release of several therapeutic molecules. The current study reports that the bone marrow mesenchymal stem cells (BMSCs) interact with NSCs *via* EVs thereby affecting the survival of neuronal cells. Hypoxic injury models of neuronal cells were established using cobalt chloride, followed by co-culture with BMSCs and NSCs alone or in combination. BMSCs combined with NSCs elicited as a superior protocol to stimulate neuronal cell survival. BMSCs-derived EVs could protect neuronal cells against hypoxic injury. Silencing of miR-133b incorporated in BMSCs-derived EVs could decrease the cell viability and the number of NeuN-positive cells and increase the apoptosis in the CA rat model. BMSCs-derived EVs could transfer miR-133b to neuronal cells to activate the AKT-GSK-3β-WNT-3 signaling pathway by targeting JAK1. Our study demonstrates that NSCs promotes the release of miR-133b from BMSCs-derived EVs to promote neuronal cell survival, representing a potential therapeutic strategy for the treatment of CA-induced brain damage.

## INTRODUCTION

Cardiac arrest (CA), as a leading cause of mortality and chronic disability worldwide, is characterized by extensive brain damage with cognitive impairment, which radically affect the quality of life [1]. Although there are great advancements in CA treatment, its mortality and morbidity rates remain alarmingly high due to the associated secondary syndromes, such as myocardial dysfunction and neurological impairment [2]. In recent years, neural stem cells (NSCs) and mesenchymal stem cells (MSCs) have exhibited potential in attenuating brain damage and improving neurobehavioral recovery by facilitating the release of numerous neurotrophic factors and cytokines, or differentiating into multiple cell types to recuperate for ischemia/reperfusion-induced cell death [3–5]. Transplantation of bone marrow MSCs (BMSCs) has also been demonstrated its significance as a promising therapy for neurological disorders [6]. Moreover, a combination protocol of MSCs-secreted factors and NSC transplantation can stimulate the functional recovery of neurons in Parkinson's disease [7]. Therefore, it is important to further investigate the mechanism of combined protocol of transplantation of BMSCs and NSCs in order to recuperate CA-induced brain damage.

Reports have documented that stem cells can release extracellular vesicles (EVs), which increased the functionality of stem cells-derived EVs as a potential therapeutic target for ischemia/reperfusion-induced hepatic and renal injuries [8, 9]. EVs are membrane vesicles originating from a diversity of mammalian cell types containing small EVs (< 200 nm) and large EVs (>200 nm) [10]. Researches support the significant impact of EVs on the functional recovery of neuronal cells [11, 12]. EVs, due to their mobile nature between cells, facilitate the transfer of several proteins, and messenger RNAs (mRNAs), and microRNAs (miRNAs) [13]. Several miRNAs, such as miR-122 and miR-21 have been reported to fundamentally function as prognostic markers in CA-induced damage [14]. A recent study verified the significance of miR-133b loaded in BMSCs-derived EVs in intracerebral hemorrhage [15]. An existing study ascertained the involvement of miR-133 in some regulatory processes, including functional recovery following spinal cord injury [16]. A prediction from the bioinformatics website in our investigation speculated that Janus kinase 1 (JAK1) was a putative target gene of miR-133b. Furthermore, JAK1 has been reported to be active in neuronal cells after cerebral infarction, thereby serving as a target for neuroprotection in the acute phase of ischemia [17], while its effects on CA-induced brain damage are still enigmatic. In the current study, the role of combined transplantation of BMSCs-derived EVs-incorporated miR-133b and NSCs in improving neuronal cell survival in CA-induced brain damage and its underlying mechanisms were explored, which may pave the way for a novel direction in the treatment of CA-induced brain damage.

## RESULTS

### Identification of BMSCs, NSCs, and BMSCs-Derived EVs

**Figure 1:**
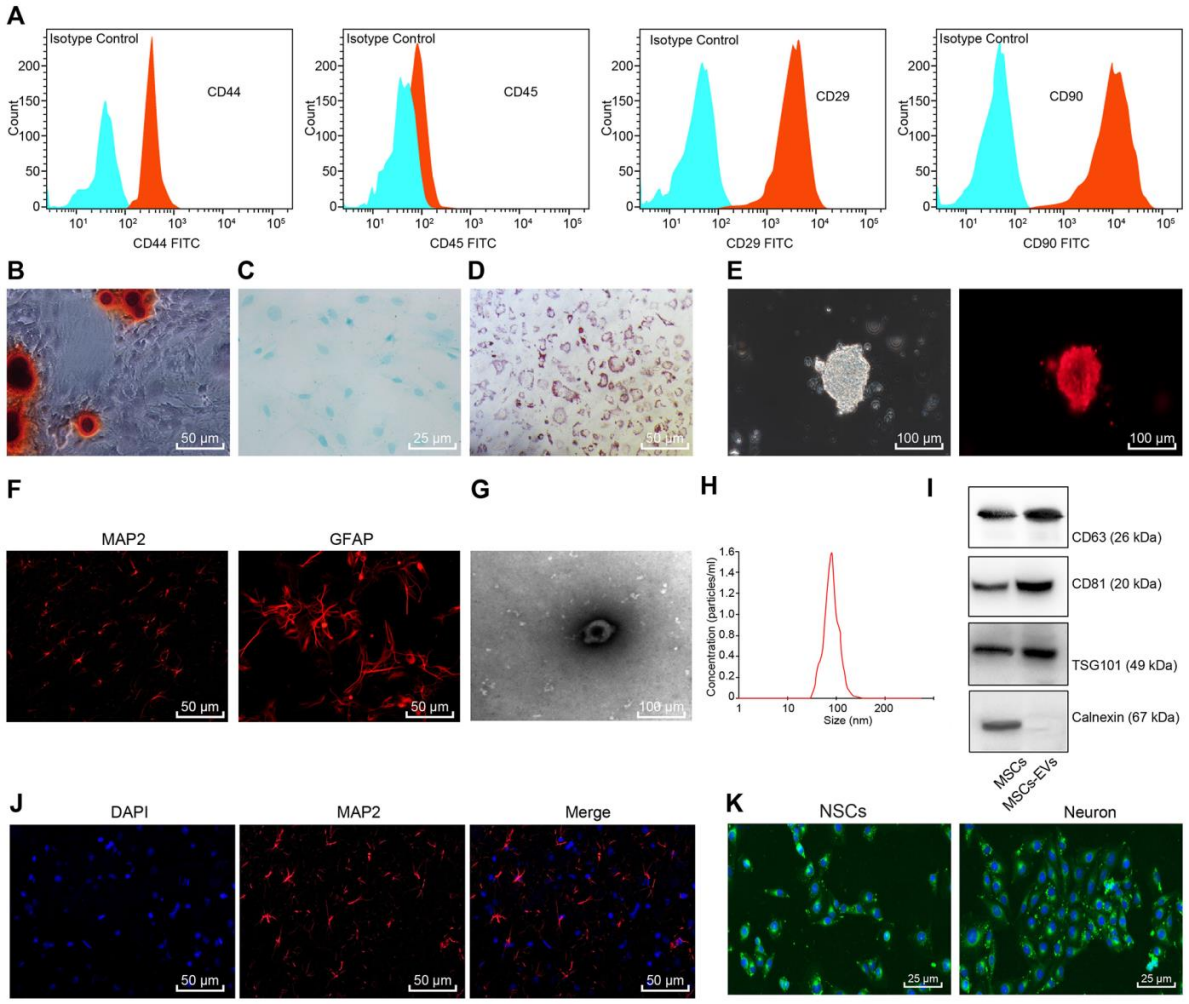
**Identification of BMSCs, NSCs, and BMSCs-derived EVs.** (**A**) Immunophenotypic analysis for BMSCs using flow cytometry. Blue represents Isotype control and red represents CD44^+^/CD45^+^/CD29^+^/CD90^+^ cells. (**B**) Alizarin red staining of BMSCs (× 200). (**C**) Alsine blue staining of BMSCs (× 400). (**D**) Oil red O staining of BMSCs (× 200). (**E**) Morphology of rat NSCs and Nestin-positive cells detected using immunofluorescence staining (scale bar = 100 μm). (**F**) The expression of MAP-2 and GFAP in the differentiated NSCs detected using immunofluorescence staining (× 200). (**G**) Morphology of EVs observed under TEM (scale bar = 100 nm). (**H**) Particle size distribution of BMSCs-derived EVs measured by NTA. (**I**) The contents of the surface markers CD81, CD63 and TSG101 in BMSCs and BMSCs-derived EVs measured using Western blot analysis. (**J**) Neuronal marker MAP-2 detected by immunofluorescence staining (× 200). (**K**) The uptake of EVs observed under the fluorescence microscope (× 400); green corresponded with the PKH-67-labelled EVs, and blue corresponded with the DAPI-stained BMSCs.

Initially, flow cytometry was performed for immunophenotypic analysis of the BMSCs, the results of which revealed that CD29, CD44, and CD90 were positive in BMSCs, while CD45 was negative (Figure 1A). After incubation in a specific differentiation medium, BMSCs could differentiate into various types of cells such as osteoblasts, adipocytes, and chondrocytes. In the osteogenesis medium, alizarin red staining showed BMSCs with a cubic representation and aggregation to form mineralized nodules (Figure 1B). Alsine blue staining was performed 21 days after induction of cartilage formation (Figure 1C). Oil red O staining exhibited red lipid droplets in the cells (Figure 1D). The neurosphere formation of rat NSCs was observed under an optical microscope (Figure 1E). Immunofluorescence staining revealed a positive Nestin expression (Figure 1E). Successful differentiation of NSCs into neuronal cells positive was observed for the microtubule-associated protein 2 (MAP-2) and glial cells positive for GFAP (Figure 1F). Hence, BMSCs and NSCs were successfully identified.

The EVs of BMSCs were separated by means ultracentrifugation and identified. The results revealed that the separated vesicles were round while elliptical membrane vesicles with discoid structure, complete envelope, and similar morphology (Figure 1G). Nanoparticle tracking analysis (NTA) showed that the particle size of BMSCs-derived EVs was about 50 - 150 nm (Figure 1H). Western blot analysis displayed that the contents of the surface markers CD81, CD63 and tumor susceptibility gene 101 (TSG101) in BMSCs-derived EVs were significantly higher compared to the BMSCs, while no expression of Calnexin was found in EVs (Figure 1I), which further demonstrated that the extraction of EVs was successful. Next, the expression profile of the neuronal cell marker MAP-2 in the isolated neuronal cells was determined using immunofluorescence staining (Figure 1J), which validated more than 90% purity of the neuronal cells. After co-culture of NSCs and neuronal cells with the PKH-67-labelled EVs for 48 h, we observed the uptake of EVs by NSCs and neuronal cells. Green fluorescence (PKH-67-labelled EVs) was detected in the NSCs and neuronal cells (Figure 1K).

### BMSCs and NSCs protect neuronal cells from hypoxia injury

**Figure 2:**
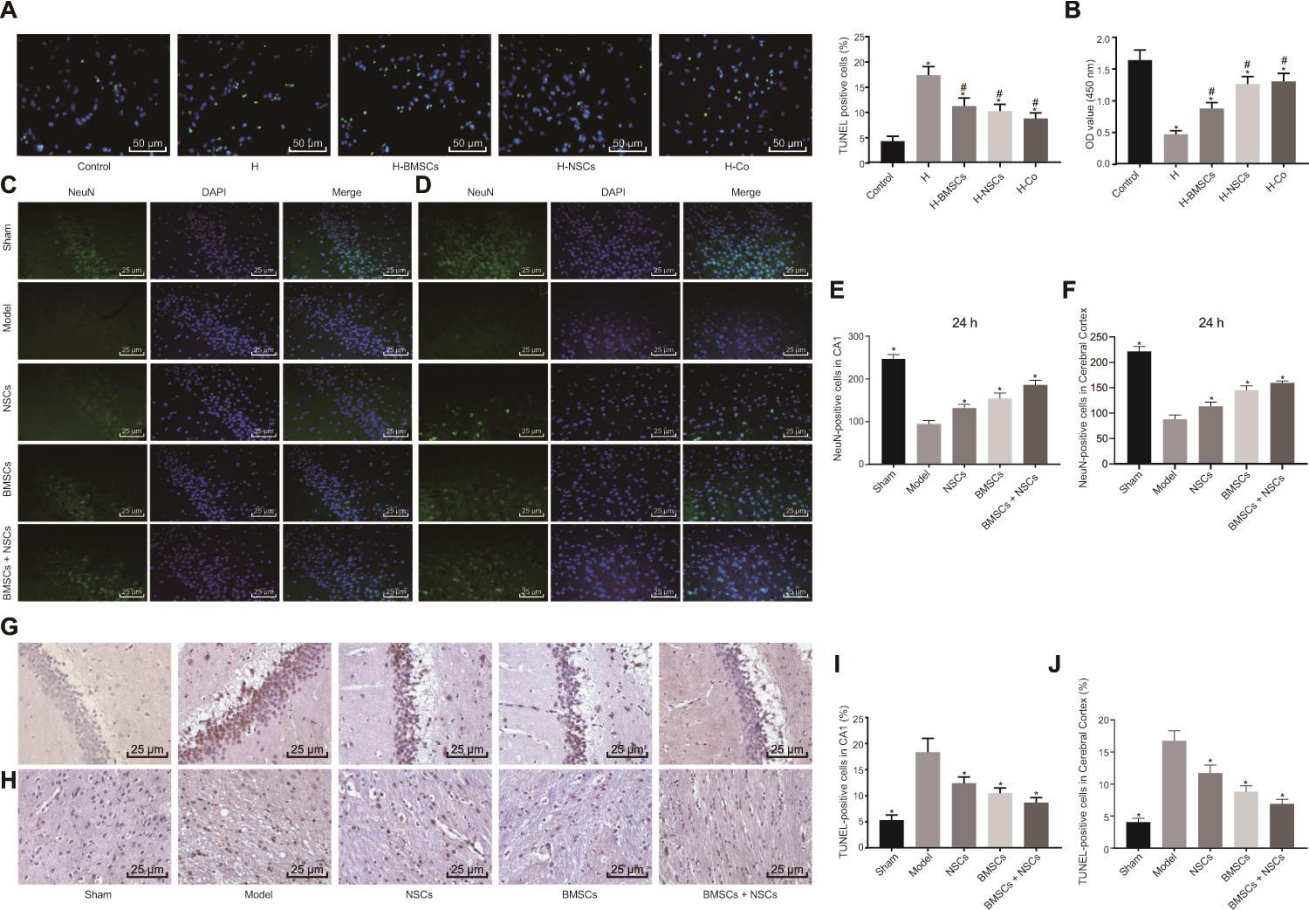
**Combined treatment of BMSCs and NSCs promotes survival of neuronal cells *in vivo* and *in vitro.*** Neuronal cells injured by hypoxia were untreated (H) or co-cultured with BMSCs (H-BMSCs), NSCs (H-NSCs) or BMSCs + NSCs (H-Co) in Panels (**A** and **B**). The sham-operated rats were not treated with any cells (sham) while CA rats were not treated (Model) or injected with BMSCs, NSCs or BMSCs + NSCs in Panels (**C**–**J**). (**A**) Apoptosis of neuronal cells assessed by TUNEL staining (× 200). (**B**) Cell viability assessed by CCK-8 assay. (**C**) Representative images of the NeuN-positive cells in the hippocampal CA1 region visualized using immunofluorescence staining (× 400). (**D**) Representative images of NeuN-positive cells in the cerebral cortex visualized using immunofluorescence staining (× 400). (**E**) The number of NeuN-positive cells in the hippocampal CA1 region. (**F**) The number of NeuN-positive cells in the cerebral cortex. (**G**) Apoptosis of neuronal cells in the hippocampal CA1 region assessed by TUNEL staining (× 400). (**H**) Apoptosis of neuronal cells in the cerebral cortex assessed by TUNEL staining (× 400). (**I**) Comparison of apoptotic rate in the hippocampal CA1 region. (**J**) Comparison of apoptotic rate in the cerebral cortex. * *p* < 0.05 *vs.* the Control (neuronal cells without any treatment) or Model (rats with CA without any treatment) group, # *p* < 0.05 *vs.* the H group (hypoxia-induced injured neuronal cells without any treatment). Data were expressed as mean ± standard deviation, and comparison among multiple groups were analyzed by one-way ANOVA followed by Tukey's post hoc test. n = 10 in animal experiments. The cell experiments were conducted 3 times independently.

Neuronal cells were treated with cobalt chloride to establish a model of hypoxia-induced cell injury *in vitro*, followed by treatment with one or two types of stem cells to observe the resulting protective effects. Neuronal cells injured by hypoxia remained untreated or were treated with BMSCs, NSCs or a combination of BMSCs and NSCs. The results of TdT-mediated dUTP Nick-End Labeling (TUNEL) (Figure 2A) and Cell Counting Kit-8 (CCK-8) (Figure 2B) assay showed suppressed viability and simulative apoptosis in neuronal cells injured by hypoxia, while treatment of BMSCs or NSCs partially inhibited cell apoptosis and facilitated cell survival. Notable results were observed in neuronal cells treated with both BMSCs and NSCs, suggesting that the combination treatment of BMSCs and NSCs together could significantly protect the neuronal cells from hypoxia-induced apoptosis.

BMSCs, NSCs alone or in combination were transplanted into the CA model rats, followed by Neurological Deficit Scoring (NDS) on the neurological function (Table 1). Results showed that treatment of NSCs, BMSCs or BMSCs + NSCs led to a significantly lower score compared to the rats without any treatment while the reduction was noteworthy in rats treated with BMSCs + NSCs, indicating that the combination transplantation of BMSCs and NSCs facilitated the recovery of cerebral injury induced by CA. Then, NeuN-positive cells at 24 h in the cerebral cortex and the hippocampal CA1 region were detected using immunofluorescence staining (Figure 2C–2F). The findings revealed that the number of NeuN-positive cells was elevated upon treatment with NSCs, BMSCs or BMSCs + NSCs, and the increase was most significant upon treatment with BMSCs + NSCs (all *p* < 0.05). TUNEL staining (Figure 2G–2J) also verified that apoptosis in the presence of BMSCs + NSCs was significantly inhibited (*p* < 0.05). Conjointly, a combination of BMSC and NSC transplantation could increase the number of NeuN-positive cells and reduce neuronal cell apoptosis in rats with CA.

**Table 1:** NDS scores of rats transplanted with NSCs and BMSCs alone or in combination.

**Time**	**Sham**	**Model**	**NSCs**	**BMSCs**	**BMSCs + NSCs**
1 h	0	197.37 ± 11.42	194.38 ± 10.24	191.38 ± 10.32	187.49 ± 9.78
24 h	0	227.24 ± 13.54	208.42 ± 11.48*	204.38 ± 11.42*	193.28 ± 10.13*
7 d	0	246.23 ± 14.53	224.14 ± 12.27*	216.28 ± 12.17*	198.76 ± 11.03*

Note: NDS, neurological deficiency score; NSCs, neural stem cells; BMSCs, bone marrow mesenchymal stem cells; * *p* < 0.05 *vs.* the Model group (rats with CA without any treatment). Data obtained from three independent experiments were expressed as mean ± standard deviation and data among multiple groups were analyzed by one-way ANOVA with Tukey's post hoc test. n = 10 for each group.

### NSCs promoted release of BMSCs-derived EVs to protect neuronal cells

**Figure 3:**
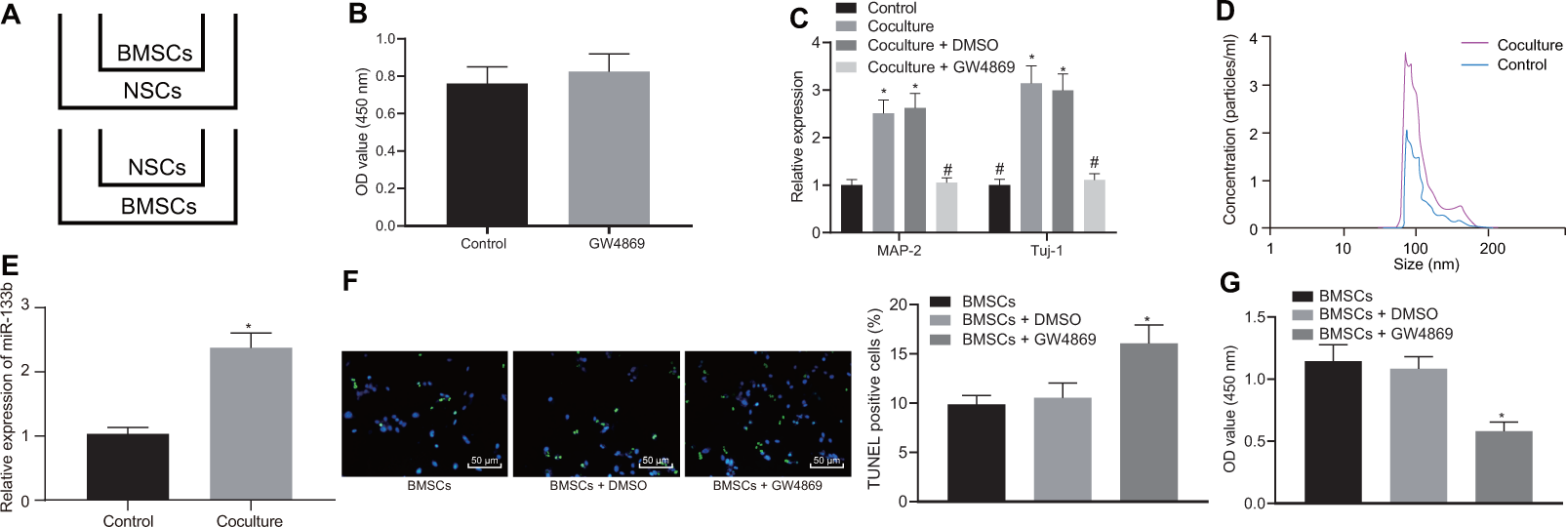
**EVs derived from BMSCs are promoted by NSCs.** The cells were grouped into Control (NSCs), Coculture (co-culture of BMSCs and NSCs), Coculture-DMSO (co-culture of BMSCs and NSCs with DMSO in the apical chamber), Coculture-GW4869 (co-culture of BMSCs and NSCs cells with GW4869 in the apical chamber). (**A**) Co-culture of BMSCs and NSCs in the Transwell chambers. (**B**) The viability of neuronal cells after GW4869 (an inhibitor for EVs) treatment determined using CCK-8 assay. (**C**) The mRNA expression of neuronal cell markers (MAP-2 and Tuj-1) in the BMSCs untreated or co-cultured with BMSCs with the addition of DMSO or GW4869 in the apical chamber determined by RT-qPCR. (**D**) The concentration of EVs determined by NTA. (**E**) The expression profile of miR-133b in BMSCs-derived EVs determined by RT-qPCR. (**F**) The neuronal cell apoptosis assessed by TUNEL assay (× 200). (**G**) The neuronal cell proliferation after co-culture with EVs detected by CCK-8 assay. * *p* < 0.05 *vs.* NSCs or BMSCs alone. # *p* < 0.05 *vs.* co-culture of BMSCs and NSCs. Data are expressed as mean ± standard deviation, and data between two groups were analyzed using the unpaired *t* test while data among multiple groups were analyzed by one-way ANOVA with the Tukey's post hoc test. The cell experiments were conducted 3 times independently.

With results eliciting the therapeutic effects of a combination of BMSCs and NSCs, we speculated an underlying interaction between BMSCs and NSCs to support their functionality. For exploration purpose, the BMSCs and NSCs were co-cultured in an indirect manner. Firstly, the effects of BMSCs on NSCs were investigated. During co-culture, NSCs placed in the basolateral chambers were co-cultured with BMSCs with addition of dimethyl sulfoxide (DMSO) or GW4869 (an inhibitor for EVs) in the apical chambers to evaluate the differentiation of NSCs (Figure 3A). Our results elicited that GW4869 treatment did not affect the viability of the cultured normal NSCs (Figure 3B). Following culture for 2 weeks, reverse transcription quantitative polymerase chain reaction (RT-qPCR) was conducted to measure the expression profiles of markers (MAP-2 and Tuj-1) (Figure 3C). Results showed that compared with the NSCs alone, co-culture of BMSCs and NSCs significantly promoted the expression of MAP-2 and Tuj-1 while the addition of GW4869 reduced the expression of MAP-2 and Tuj-1, suggesting that BMSCs contributed to the differentiation of NSCs into neuronal cells depending on the release of EVs. Subsequently, the effects of NSCs on BMSCs were explored. During co-culture, BMSCs were placed in the basolateral chambers with/without the addition of NSCs. The culture medium of BMSCs was collected to extract the EVs, followed by a regimen NTA (Figure 3D). Results revealed the release of EVs from BMSCs and a significantly higher concentration of EVs in the presence of NSCs, indicating an interaction between BMSCs and NSCs. In addition, our results revealed that the NSCs lead to an upregulated miR-133b in BMSCs-derived EVs (Figure 3E).

Since the co-culture of BMSCs and NSCs facilitated the release of EVs from BMSCs and the combination treatment facilitated the survival of neuronal cells of rats with CA, we endeavored to ascertain whether the stimulating effects were dependent on the increase of EV release. Herein, BMSCs were co-cultured with neuronal cells under hypoxic conditions to observe the relation between the release of BMSCs-derived EVs and neuronal cell survival. As shown in Figure 3F, 3G, in response to GW4869, the cell apoptosis was elevated while the cell survival was suppressed according to the results of TUNEL assay and CCK-8 assay. These findings demonstrated that BMSCs-derived EVs conferred the protection on neuronal cell survival after exposure to hypoxia.

### miR-133b in EVs derived from BMSCs promoted survival of neuronal cells to improve neurological functions of rats with CA

**Figure 4:**
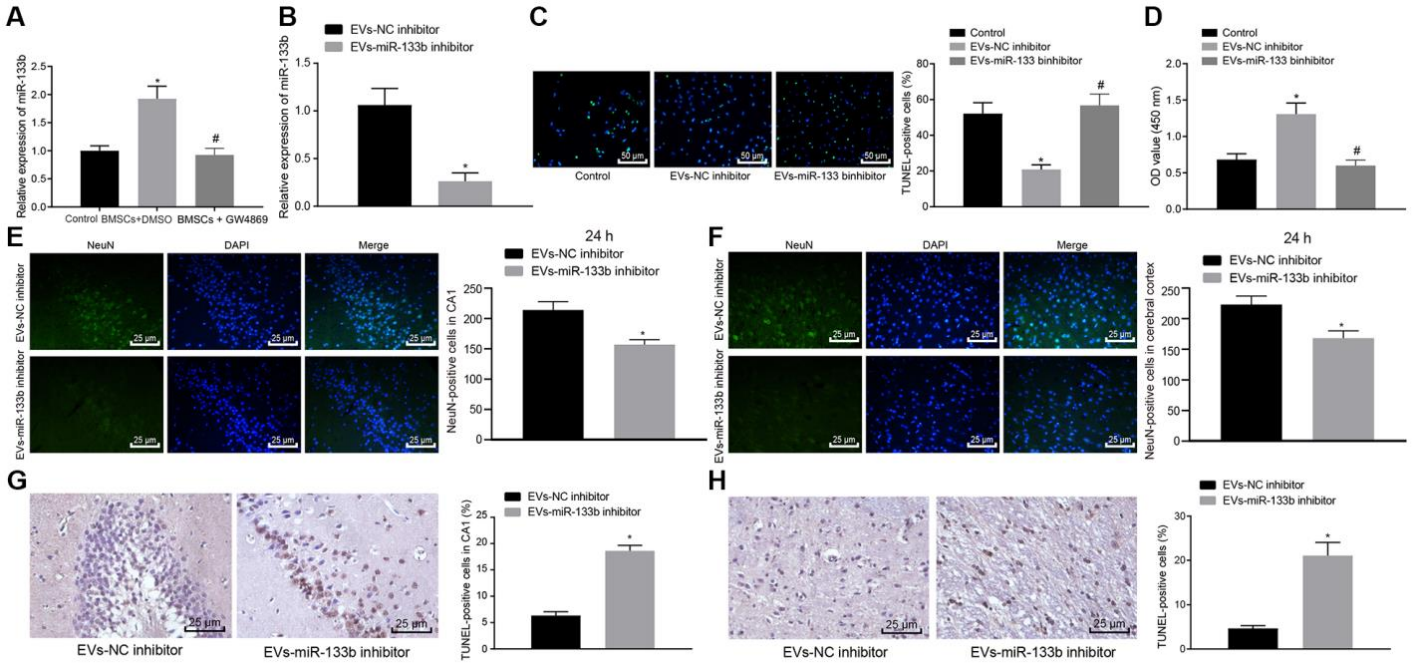
**Neuronal cell survival in rats with CA is promoted by miR-133b transferred *via* BMSCs-derived EVs.** (**A**) The miR-133b expression in neuronal cells following co-culture with BMSCs and treatment of GW4869 or DMSO determined by RT-qPCR. (**B**) The miR-133b expression in neuronal cells following co-culture with EVs derived from the BMSCs treated with miR-133b inhibitor determined by RT-qPCR. (**C**) The neuronal cell apoptosis detected by TUNEL assay (× 200). (**D**) The neuronal cell proliferation detected by CCK-8 assay. (**E**) The number of NeuN-positive cells in hippocampal CA1 region (× 400). (**F**) The number of NeuN-positive cells in cerebral cortex (× 400). (**G**) Apoptosis of neuronal cells in hippocampal CA1 region assessed by TUNEL staining (× 400). (**H**) Apoptosis of neuronal cells in cerebral cortex assessed by TUNEL staining (× 400). * *p* < 0.05 *vs.* the EVs-NC inhibitor group (neuronal cells co-cultured with EVs derived from the BMSCs treated with NC inhibitor). Data were expressed as mean ± standard deviation. Data between two groups were analyzed by unpaired *t* test while data among multiple groups were analyzed by one-way ANOVA with Tukey's post hoc test. n = 10 in animal experiments. The cell experiments were conducted 3 times independently.

With the aforementioned findings ascertaining that the co-culture of NSCs and BMSCs could promote the expression of miR-133b in EVs derived from BMSCs and EVs could be protective of the neuronal cells, we hypothesized that miR-133b shuttled by EVs might be functional. RT-qPCR was performed to determine the expression profile of miR-133b in neuronal cells after co-culture with BMSCs (Figure 4A). Results revealed markedly up-regulated level of miR-133b in neuronal cells co-cultured with BMSCs and treated with DMSO compared with the untreated neuronal cells, whereas a significantly lower miR-133b expression was observed in response to GW4869 treatment in the neuronal cells co-cultured with BMSCs, suggesting that EVs might serve as a conveyance of miR-133b to the neuronal cells. Next, EVs derived from the BMSCs infected with the NC inhibitor or miR-133b inhibitor were co-cultured with the neuronal cells. As detected by means of RT-qPCR (Figure 4B), TUNEL assay (Figure 4C) and CCK-8 assay (Figure 4D), treatment with EVs derived from the NC inhibitor-treated BMSCs markedly stimulated the survival and inhibited the apoptosis of neuronal cells compared to the untreated neuronal cells. Additionally, the miR-133b expression was diminished in response to treatment with EVs derived from the miR-133b inhibitor-treated BMSCs accompanied by suppressed neuronal cell survival and promoted apoptosis. The following experiments were conducted to investigate validate the effects of miR-133b in EVs derived from BMSCs on cell apoptosis *in vivo*. EVs were extracted from BMSCs infected with NC inhibitor or miR-133b inhibitor and then injected in both ventricles of rats with CA. Results of immunofluorescence assay (Figure 4E, 4F) and TUNEL assay (Figure 4G, 4H) showed that NeuN-positive cells at 24 h in the cerebral cortex and hippocampal CA1 region significantly decreased after treatment with EVs derived from the miR-133b inhibitor-treated BMSCs along with increased cell apoptosis. Altogether, these results confirmed the functional role of miR-133b in EVs derived from BMSCs.

### miR-133b in EVs derived from BMSCs targeted JAK1 to Activate the AKT-GSK-3β-WNT pathway

**Figure 5:**
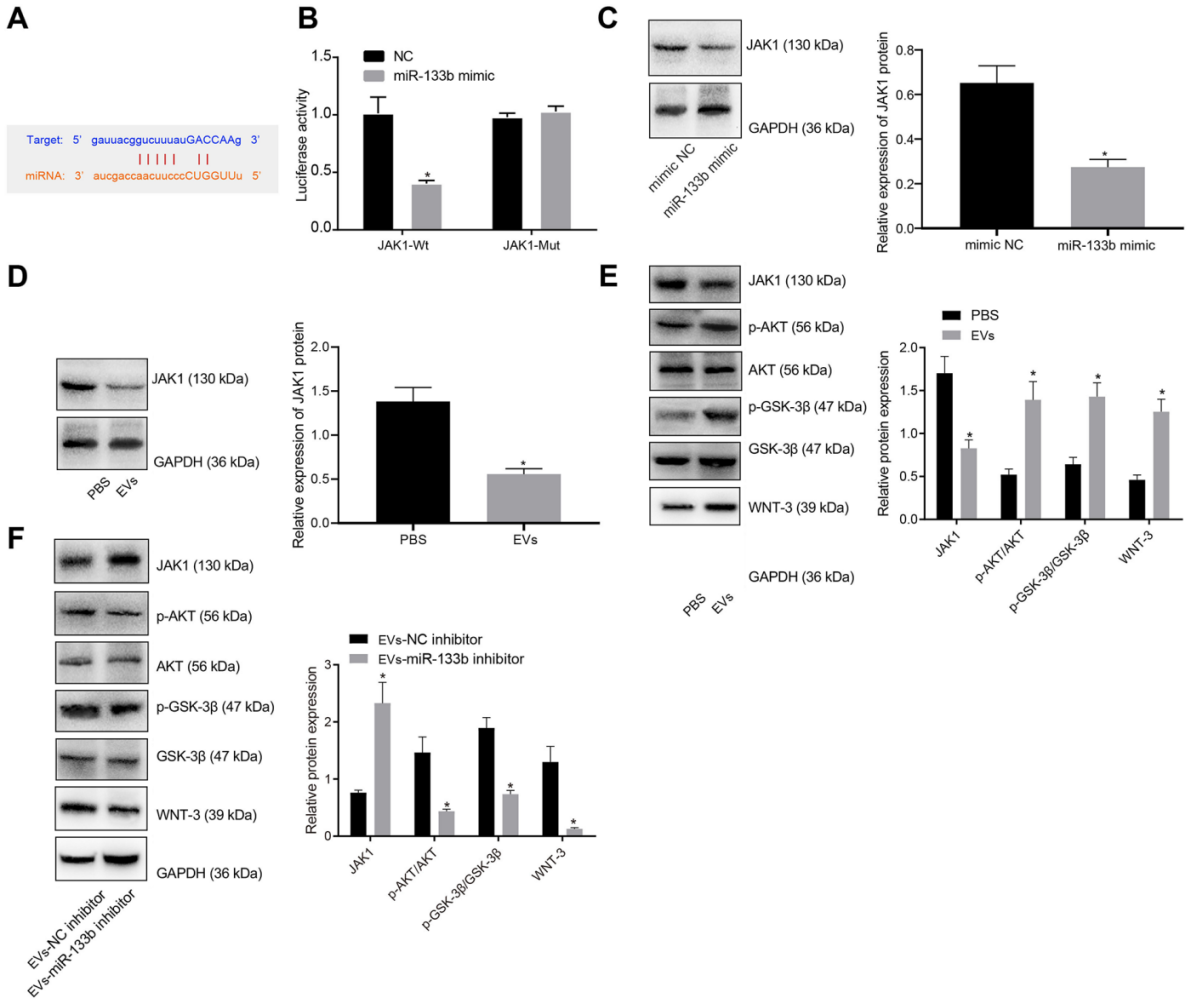
**miR-133b in BMSCs-derived EVs mediates the AKT-GSK-3β-WNT pathway by targeting JAK1.** (**A**) Prediction binding site of miR-133b in JAK1 3'-UTR. (**B**) Detection of luciferase activity using dual-luciferase reporter gene assay; * *p* < 0.05 *vs.* the NC group. (**C**) The expression of JAK1 in neuronal cells normalized to GAPDH in response to miR-133b mimic determined by Western blot analysis; * *p* < 0.05 *vs.* the mimic NC group (neuronal cells treated with mimic NC). (**D**) The expression of JAK1 in neuronal cells normalized to GAPDH in response to treatment of BMSCs-derived EVs determined by Western blot analysis. (**E**) Protein levels of JAK1, p-AKT/AKT, p-GSK-3β/GSK-3β, and WNT-3 in the neuronal cells normalized to GAPDH in response to treatment of BMSCs-derived EVs determined by Western blot analysis. * *p* < 0.05, *vs.* the PBS group (neuronal cells treated with PBS). (**F**) Protein levels of JAK1, p-AKT/AKT, p-GSK-3β/GSK-3β, and WNT-3 in the neuronal cells normalized to GAPDH in response to treatment of EVs derived from the BMSCs treated with miR-133b inhibitor determined by Western blot analysis. * *p* < 0.05, *vs.* the EVs-inhibitor NC group (neuronal cells in co-culture system with EVs derived from the BMSCs treated with inhibitor NC). Data obtained from three independent cell experiments were expressed as mean ± standard deviation. Data between two groups were analyzed by the unpaired *t* test.

The bioinformatics database presented with specific binding sites between JAK1 and miR-133b (Figure 5A), which was subsequently verified by means dual-luciferase reporter gene assay (Figure 5B). The results showed that the luciferase activity of wild type (WT)-miR-133b/JAK1 was significantly inhibited by the miR-133b mimic (*p* < 0.05), while no difference was evident in the mutant type (MUT)-JAK1 3’-untranslated region (3’-UTR) (*p* > 0.05). The relation between miR-133b and JAK1 was further investigated in the neuronal cells, the results of which revealed an inverse relation supporting that miR-133b mimic significantly downregulated the expression of JAK1 (Figure 5C), Likewise, EVs derived from BMSCs significantly inhibited the expression of JAK1 in neuronal cells (Figure 5D). Additionally, Western blot analysis for quantification of JAK1 and the downstream factors of the AKT-GSK-3β-WNT pathway showed elevated phosphorylated-protein kinase B (p-AKT)/AKT and phosphorylated-GSK-3β (p-GSK-3β)/GSK-3β ratios and WNT-3 protein level, but reduced JAK1 protein level in the neuronal cells cultured with EVs in comparison to the neuronal cells treated with PBS (*p* < 0.05) (Figure 5E). After transduction with the NC inhibitor or miR-133b inhibitor in BMSCs, EVs were extracted for co-culture with the neuronal cells. Further Western blot analysis (Figure 5F) revealed reduced p-AKT/AKT and p-GSK-3β/GSK-3β ratios and WNT-3 protein level, but elevated JAK1 protein level in response to a lower proportion of miR-133b transferred by EVs. The aforementioned findings elucidated that miR-133b in BMSCs-derived EVs could activate the AKT-GSK-3β-WNT pathway by targeting JAK1.

### miR-133b protects neuronal cells by targeting JAK1

**Figure 6:**
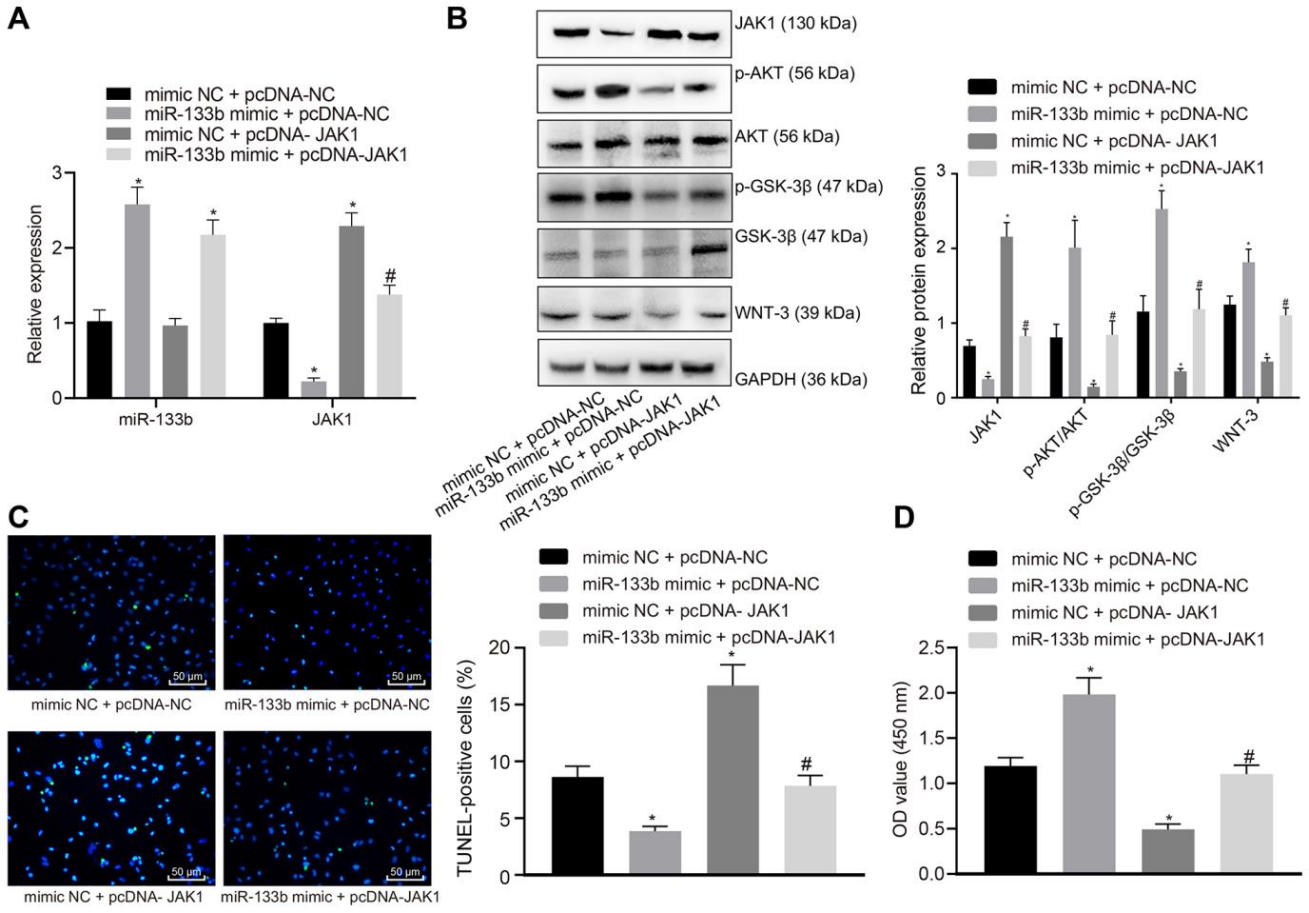
**miR-133b confers protection against neuronal cell apoptosis induced by hypoxia through inhibition of JAK1.** (**A**) miR-133b expression profile and JAK1 mRNA level determined using RT-qPCR. (**B**) Protein levels of JAK1, p-AKT/AKT, p-GSK-3β/GSK-3β, and WNT-3 normalized to GAPDH determined using Western blot analysis. (**C**) Apoptosis of neuronal cells assessed by TUNEL staining (× 200). (**D**) Cell viability detected by CCK-8 assay. * *p* < 0.05 *vs.* the mimic NC + pcDNA-NC group (neuronal cells treated with mimic NC + pcDNA-NC); # *p* < 0.05 *vs.* the miR-133b mimic + pcDNA-NC group (neuronal cells treated with miR-133b mimic + pcDNA-NC). Data obtained from three independent cell experiments were expressed as mean ± standard deviation and data among multiple groups were analyzed by one-way ANOVA followed by the Tukey's post hoc test.

Following treatment with cobalt chloride, miR-133b mimic and pcDNA-JAK1 were transduced into the neuronal cells. RT-qPCR (Figure 6A) revealed that in the presence of pcDNA-NC, miR-133b expression was elevated while the JAK1 expression was reduced by addition of miR-133b mimic compared to mimic NC while upregulated JAK1 expression was observed in the presence of mimic NC + pcDNA-JAK1. In the presence of miR-133b mimic, the addition of pcDNA-JAK1 restored the JAK1 expression. Further Western blot analysis (Figure 6B), TUNEL assay (Figure 6C) and CCK-8 assay (Figure 6D) showed that the enhancement of miR-133b resulted in elevations in p-AKT/AKT and p-GSK-3β/GSK-3β ratios, WNT-3 protein level and survival rate as well as a reduction in the apoptosis rate. However, restoration of the JAK expression decreased the p-AKT/AKT and p-GSK-3β/GSK-3β ratios as well as WNT-3 protein level corresponding to a lower survival rate and a higher apoptosis rate. Furthermore, the effects of miR-133b mimic were evidently counteracted by pcDNA-JAK1. To conclude, miR-133b exerted vital protective effects on the survival of neuronal cells following the induction of hypoxia through inhibition of JAK1.

## DISCUSSION

CA is a prevalent fatal disease affecting millions of people around the world on an annual basis, and for which the effective therapeutic target for its treatment is under development [18]. A combination protocol of BMSC-secreted factors and NSC transplantation have been reported to stimulate the functional recovery of neurons in rats with Parkinson's disease [7], while their effects on CA-induced brain impairment need extensive research. Our study aimed at exploring the promising effect of BMSCs harboring restored miR-133b in EVs and NSCs on neuronal cell survival in CA-induced brain damage. Conjointly, the current study reveals that NSCs and BMSCs-derived EVs-loaded miR-133b can facilitate the neuronal cell survival *via* regulation of JAK1 and AKT-GSK-3β-WNT pathway to mitigate the CA-induced brain damage.

Initially, we validated that the combination protocol of BMSCs and NSCs transplantation *in vitro* inhibited neuronal cell apoptosis and promoted their survival, thereby protecting them against cobalt chloride-induced damage. Stem cell transplantation has served as a viable way of cell replacement therapy. A study cited the ability of approximately 1/3 of stem cells to migrate to the damaged region upon local or systematic injection [19]. A recent study demonstrated that BMSCs could facilitate the differentiation ability of NSCs and furthermore protect against oxidative stress injury-induced apoptosis of NSCs [20, 21]. An existing study also highlighted the simulative effects of bone marrow-derived mesenchymal stromal stem cells on the stemness of NSCs [22]. Research has elicited the association between BMSCs and NSCs to facilitate the proliferation and differentiation of NSCs *via* mediation of the notch-signaling pathway [23]. Meanwhile, NSCs could potentially induce BMSC differentiation into neural stem-like cells [24]. Although our study demonstrated that the transplantation of both BMSCs and NSCs into CA rats could impede the apoptosis of neuronal cells, however these findings warrant further investigation.

In a subsequent experiment, our results demonstrated that miR-133b was enriched in BMSCs-derived EVs and could be transferred to NSCs *via* EVs. A recent study has demonstrated the ability of MSCs to secrete EVs containing multiple miRNAs that can be transferred to cells, and miR-223 is highly expressed in BMSCs [25]. Notably, interaction between BMSCs and NSCs has been documented to fundamentally exercise the simulative effects on differentiation of BMSCs into NSCs and neuron cells [26]. Additionally, miR-133b has been proven to be delivered to the neurons *via* EVs secreted from MSCs [15]. Moreover, Shen et al*.* have revealed the capacity of miR-133b to be shuttled to the brain tissues as a cargo of MSCs-derived EVs in rats after intracerebral hemorrhage [27]. Consistent with these findings, our data revealed that BMSCs could shuttle miR-133b to NSCs to exert protective effects for neuron cell survival.

Furthermore, our findings elucidated that silencing of BMSCs-derived EVs-incorporated miR-133b could inhibit cell viability and the number of NeuN-positive cells, and increase apoptosis, and also validated that BMSCs-derived EVs-incorporated with miR-133b ameliorated the CA-induced cell apoptosis. An existing study ascertained that EVs derived from astrocytes could stimulate neuronal cell survival in the hypoxic and ischemic environment [28]. MSCs-derived EVs-loaded overexpressing miR-133b to neural cells can augment neurite outgrowth [15]. Notably, the delivery of miR-133b *via* EVs secreted from MSCs can inhibit the apoptosis of neural cells so as to employ the neuroprotective effects following an intracerebral hemorrhage [27]. Also, EVs from MSCs could evidently mediate the transfer of miR-133b to neuronal cells to support the functional recovery of rats after stroke [29]. In consistency with our results, an elevated level of miR-133b could fundamentally prevent the apoptosis of hippocampal neuronal cells in rats with depression [30]. Yu et al*.* have also demonstrated that overexpression of miR-133b aids to the inhibition of neuronal cell apoptosis and the regeneration of neurons in brain injury [16]. Herein, it is viable to select BMSCs delivering miR-133b *via* EVs as great therapeutic targets in respect to their potential for neuronal cell survival in CA-induced brain damage.

In addition, our experimental data elucidated JAK1 as a target gene of miR-133b. BMSCs-derived EVs-loaded miR-133b could inhibit the JAK1 expression and activate the AKT-GSK-3β-WNT signaling pathway. JAK1 is regarded as a direct target of miR-340, which consequently negatively regulates JAK1 [31]. Additionally, miR-708 can inhibit the JAK1 expression to facilitate neuronal cell survival [17]. Accumulating evidence has implied that the PI3K-AKT signaling pathway participates in mediation of neuronal cell apoptosis in the brain, and GSK-3β, a critical downstream protein of AKT, is also implicated in the pathological progression of brain damage [32]. An existing study ascertained the functionality of the PI3K-AKT-GSK3β signaling pathway to promote neuronal cell proliferation and differentiation to improve neuronal cell function [33]. Similarly, miR-27a could negatively regulate the GSK-3β expression and WNT pathway [34]. Our findings suggested that miR-133b protected neuronal cells from cobalt chloride-induced apoptosis by targeting JAK1. However, additional evidence is warranted to establish significance of the AKT-GSK-3β-WNT signaling pathway in this protective mechanism in future studies.

**Figure 7:**
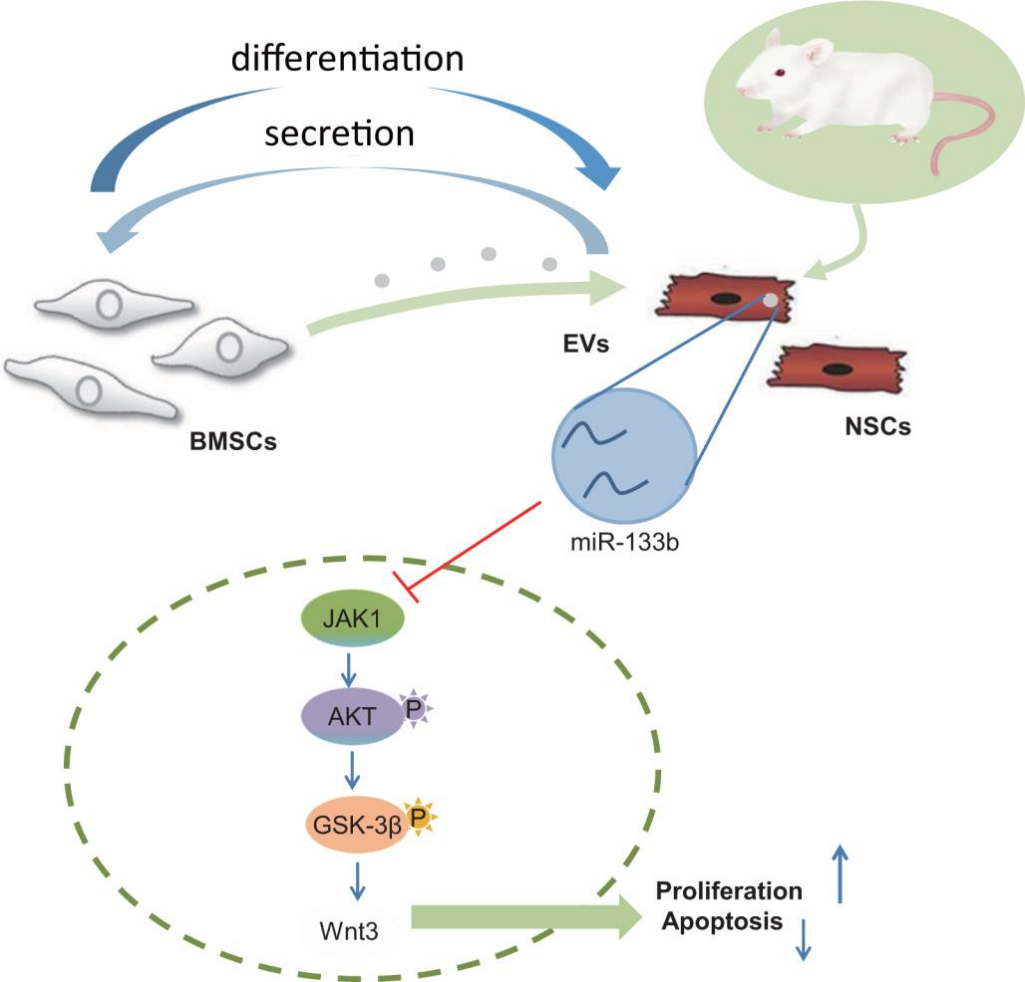
**Systematic diagrams sketching the protection of combined transplantation of BMSCs and NSCs in CA-induced neurological impairment.** BMSCs promote NSC differentiation into neuronal cells and NSCs stimulate BMSCs to release EVs harboring miR-133b into the neuronal cells thereby facilitating neuronal cell survival.

In conclusion, our findings have proved that EVs from BMSCs can shuttle miR-133b into NSCs to amplify neuronal cell survival (depicted in Figure 7). Thus, a combination of BMSCs-derived EVs-incorporated miR-133b and NSC transplantation may serve as a promising new insight for developing therapeutic treatments for CA-induced brain damage. However, the research is still in the preclinical stage, and the investigation on the mechanism of action is insufficient. Further experiments are warranted to omit the effects of stem cell differentiation on increased neuronal cell number.

## MATERIALS AND METHODS

### Ethics statement

Animal experiments were conducted in strict accordance with the Guide for the Management and Use of Laboratory Animals issued by the National Institutes of Health. The animal experiment protocol was conducted with approval of the Animal Ethics Committee of Kunming Medical University. Adequate measures were taken to minimize the suffering of animals included in this study.

### Isolation and culture of NSCs

The post-implantation (13.5 dpc) embryos were isolated from pregnant female Sprague Dawley (SD) rats after which the hippocampus was dissected in phosphate buffer saline (PBS, pH = 7.4, Gibco BRL, Carlsbad, CA, USA) mechanically. After a regimen of centrifugation at 1000 rpm for 5 min, the NSCs were suspended in Dulbecco’s modified Eagle’s medium (DMEM)/F12 (supplemented with 20 μg/mL epidermal growth factor, 20 μg/mL basic fibroblast growth factor, 1% B27, 2 mM/L glutamine, 5 IU penicillin and 5 μg/mL streptomycin). The collected NSCs were plated in 25 cm^2^ flasks (2 × 10^8^/L), and half of the liquid volume was replaced every 2 - 3 days. NSCs were passaged every 5 - 7 days and employed in experimentation after three consecutive passages. The cell morphology and growth condition were observed under an optical microscope. The single-cell suspension was applied to the coverslips. Immunofluorescence staining was performed to detect the expression of Nestin. The NSCs were detached using 0.25% trypsin containing 0.02% ethylenediaminetetraacetic acid (EDTA). The aforementioned reagents were purchased from Gibco. To determine whether NSCs could successfully differentiate into neuronal cells and glial cells, induction reagents were purchased from CyaGen Biotechnology Co., Ltd. (MUXNX-90081 and MUXNX-90091; Changsha, Hunan, China). Immunofluorescence was conducted to determine the expression of the neuronal cell marker (MAP-2), and the mature glial cell marker glial fibrillary acidic protein (GFAP).

### Isolation and identification of BMSCs

The bone marrow was mechanical dissociated from SD rats. The bone marrow was harvested using serum-free low glucose (LG)-DMEM (Sigma, St. Louis, MO, USA). The samples were then gently transferred onto a Ficoll density gradient (Sigma) at a ratio of 1: 3 and centrifuged at 300 ×g for 20 min. The cells were collected and transferred into new centrifugal tubes, and rinses with LG-DMEM (Sigma) supplemented with 10% fetal bovine serum (FBS; Hyclone, Logan, Utah, USA) two times. The cells were cultured in LG-DMEM supplemented with 100 U/mL penicillin and 100 μg/mL streptomycin with 5% CO_2_ at 37° C. After three days, the adherent cells were regarded as BMSCs at passage 0 (P0 BMSCs). Upon attaining 80-90% cell confluence, the cells were passaged or collected for subsequent injection. P3-4 BMSCs were applied for lentiviral infection.

Then MSCs were cultured using the OriCell MSC medium for osteogenesis, adipogenesis or cartilage differentiation (Cyagen Biosciences Inc., Guangzhou, China), respectively, according to the provided instructions. The MSCs were stained with alizarin red, alsine blue and oil red O staining, respectively. After 2 rinses with phosphate buffer saline (PBS), the MSCs were detached using 0.25% trypsin and adjusted to 10^5^ cells/mL. After culture with the fluorescence-labelled antibodies against rat cluster of differentiation 29 (CD29)/CD90/CD44/CD45 (Abcam Inc., Cambridge, MA, USA), the cells were incubated in dark conditions at 4° C for 30 min. After centrifugation at 200 ×g, the cell fragments were resuspended using PBS. The flow cytometer was employed to identify the surface markers of BMSCs (CD29, CD44, CD45, and CD90) (Beckton Dickinson, BD Bioscience, San Jose, CA, USA).

### Co-culture of BMSCs and NSCs

The Transwell chambers were adopted for co-culture of BMSCs and NSCs to determine the cell-cell communication between BMSCs and NSCs during differentiation. Briefly, BMSCs or NSCs were seeded onto the 6-well Transwell (with 0.4 μm pores; Millipore Corp., Bedford, MA, USA) at a density of 5 × 10^5^ cells/ml and cultured using the different media at 5% CO^2^ and 37° C for a week. The NSCs were cultured using DMEM/F12 (1: 1) supplemented with N2 (1: 50), and B27 (1: 100) (Invitrogen, Life Technologies, Carlsbad, CA, USA).

### Isolation and treatment of neuronal cells

SD pregnant rats after gestation for 13.5 days were euthanized by instilling an overdose of CO_2_, followed by collection of the embryos with sterilized devices. The hippocampus was immediately isolated from the brains of the embryos at a low temperature, rinsed with pre-cold PBS and detached using 0.25% trypsin at 37° C for 10 min. After terminating the detachment, the tissues were prepared into a homogenate. The supernatant was removed through centrifugation. DMEM/F12 supplemented with 20% FBS was added for inoculation. After 6 h, the neuronal cells unable to adhere to the coverslip were discarded. The remaining cells were seeded in the culture plate and cultured with cytosine (5 μg/mL) on the following day. The neuronal cells were obtained and cultured to the logarithmic growth phase. A hypoxia model was developed through treatment with 400 μmol/L of cobalt chloride for 24 h.

### Lentiviral infection

The recombinant lentivirus vector LV-1 (pGLVU6/GFP) with miR-133b inhibitor (LV-anti-miR-133b) or miR-133b inhibitor negative control (LV-NC) were purchased from Shanghai GenePharma Co., Ltd. (C06001; Shanghai, China). The cells were infected with the lentiviral vectors according to the provided instructions. Briefly, after a 24 h regimen of incubation in the normal medium, the cells were cultured using the medium containing Polybrene (Santa Cruz Biotechnology, Santa Cruz, CA, USA). Next, the cells (5 × 10^6^ cells/well) were infected with the lentivirus at the dose of 0.5 × 10^5^ plaque-forming unit overnight for 24 h. After incubation in the renewed medium without Polybrene for 24 h, the clone with the stable expression of miR-133b inhibitor was selected using puromycin dihydrochloride (Santa Cruz Biotechnology). For overexpression of JAK1 and miR-133b, Lipofectamine^TM^ 2000 (Life Technologies, Carlsbad, CA, USA) was employed to transduce 0.5 mg pcDNA3 JAK1 and miR-133b mimic into the cells. Upon attaining the cell density of 1 × 10^6^ cells/well, the lentivirus infection was conducted. Then, 48 h later, the transduction efficiency was verified by RT-qPCR and Western blot analysis.

### Isolation and identification of EVs

EVs were isolated from the supernatant of BMSCs in the logarithmic growth phase. Initially, the cells were centrifuged at 500 ×g for 10 min at 4° C. The supernatant was extracted and then centrifuged at 2000 ×g for 30 min to eliminate the cell debris. Next, the supernatant was passaged through a 220-nm filter, and centrifuged at 100,000 ×g for 90 min. After removal of the supernatant, the precipitate was EVs. The washing sediment was resuspended using sterilized PBS, and centrifuged at 100,000 ×g at 4° C for 60 min to remove the supernatant to prevent protein contamination. After rinsing, suspension, and precipitation, the samples were resuspended using PBS, and passaged through a 220-nm filter for sterilization, and preserved at -20° C. The EVs were characterized by Western blot analysis with 3 EVs-specific biomarkers (CD81, CD63, and TSG101), and endoplasmic reticulum stress marker Calnexin. EVs were fixed using 1% glutaraldehyde, transferred to the carbon-coated copper mesh and stained with 1% phosphotungstic acid, followed by observation under the JEM-2100 transmission electron microscope (TEM) (JEOL, Tokyo, Japan). LSM images were documented using the PARTICLEMEIRIX system. NTA was performed in the NanoSight NS300 system (Malvern Instruments, Malvern, UK).

### Internalization of EVs by NSCs and neuronal cells

For the fluorescence labeling of EVs, PKH-67 (4 mg/mL, Molecular Probes, MA, USA) solution was diluted at a ratio 1 : 200 using 1 × PBS and incubated with the cells in strict accordance with the provided instructions. Then PBS was added for dissolution of the isolated EVs. Finally, the PKH-67-labeled EVs were re-suspended in saline. The mixture was then subjected to centrifugation at 100,000 ×g for 2 h at 4° C to harvest the EVs when the sucrose density ranged between 1.13 and 1.19 g/mL, followed by collection of the EVs. Before cell seeding, the plate was pre-treated with poly-lysine (Sigma). Then NSCs were incubated with the neuronal cells (1 × 10^6^ cells/well) and PKH-67-labeled EVs for 24 h. A fluorescence microscope (BX51, Olympus, Tokyo, Japan) was used for observation.

### TUNEL staining

Neuronal cells were seeded on the Poly-L-Lysine-coated slides in 24-well plates at the density of 4 × 10^5^ cells/cm^2^. Then the cells underwent fixation in 4% paraformaldehyde for 15 min. After a rinse with PBS, the fixed cells were permeabilized with 0.2% Triton X-100. Cells were equilibrated for 10 min at room temperature using the equilibration buffer. The reaction mixture containing the fluorescein-labeled nucleotide mixture and the terminal deoxynucleotidyl transferase was prepared and incubated with the cells for 1 h at 37° C in a humidified chamber. Then the reaction was terminated and the cells were rinsed with 2 × chloride-sodium citrate buffer to terminate the reaction. After the nuclei were counterstained with Hochest, the slides were mounted and observed under a fluorescence microscope (Ti-E, Nikon, Tokyo, Japan).

### CCK-8 assay

The CCK-8 reagent kit (Dojindo Laboratories, Kumamoto, Japan) was utilized to detect the cell viability. Briefly, the neuronal cells were seeded in 96-well plates at the density of 1 × 10^4^ cells/well. After incubation with 5% CO_2_ at 37° C, the cells were supplemented with the CCK-8 solution. After a subsequent 4 h regimen of incubation, the optical density (OD) was detected at the excitation wavelength of 450 nm. Cell viability (%) = OD in the experimental group/OD in the control group × 100%.

### Establishment rat models of CA

The 3-week old male SD rats weighting approximately 300 g were randomly grouped. Ten rats were anesthetized and sham-operated using the indwelling catheter while the remaining rats were used to establish CA. Rats were injected with 10% pentobarbital intraperitoneally (0.3 mL/100 g). The tube was then secured with a lip suture. The femoral artery was cannulated with the PE-50 pipe. Vecuronium bromide (1 mg/kg) was administered *via* the femoral vein. The endotracheal tube was closed at the end of expiration to terminate any mechanical ventilation. The alterations in the blood pressure of rats were observed and documented. CA establishment was definitive when the mean arterial pressure (MAP) was below 30 mm Hg. After establishing CA for 8 min, the chests were compressed *via* the standardized thumper at a rate of 200/min, a depth of 1/3 of anterior–posterior chest diameter, and at a compression/relaxation ratio of 1 : 1. Animals were ventilated with 21% O_2_ at a rate of 70%/min (tidal volume: 0.65 mL/100g) during resuscitation. O_2_ concentration and ventilation were adjusted in strict accordance with the obtained arterial blood samples. Adrenaline (2 μg/100 mg) administration and chest compressions were performed simultaneously. If necessary, the actions were repeated every 2 min. After 2 min of cardiopulmonary resuscitation (CPR), the electrocardiography was conducted to monitor the recovery. Then, defibrillator (5J) was employed for suspected ventricular fibrillation, while CPR was repeated if no obvious effects were observed. Rats capable of restoring the supraventricular rhythm with MAP > 60 mm Hg for more than 10 min were indicative of successful CPR. If return of the spontaneous circulation (ROSC) (MAP > 40 mm Hg) was not achieved 15 min after resuscitation, CPR was considered to be futile and the rats were subsequently excluded.

### Combined transplantation of BMSCs and NSCs

After ROSC for 20 min, the rats were injected with 10% pentobarbital sodium intraperitoneally (0.3 mL/100 g). The co-cultured BMSCs and NSCs (1 × 10^6^ cells/well) or BMSCs-derived EVs (200 μg; precipitated in 0.2 mL PBS) were injected into the right and left lateral ventricles of rats with CA with a micro-injector at a rate of 1 μL/min (5 μL). The needle was held in the lateral ventricle for 5 min after the injection. The room temperature was maintained at 25 ± 0.5° C during the experiment. Each group comprised of 10 rats.

### NDS

NDS was performed to assess the neurological function before establishment of CA rat models, before transplantation and at 1 h, 24 h, and 7 d post-transplantation, including 7 parameters which were as follows: consciousness, breathing, corneal re-buckling, brain reflex, auditory reflex, righting reflex, and motor function, according to the NDS system (0 - 500 grade; 0 referred to normal and 500 referred to death or brain death) by several researchers who were blinded to the study.

### Immunofluorescence staining

The frozen sections of the cerebral cortex and hippocampus were permeabilized in PBS containing 0.3% Triton X-100 for 10 min, and then subsequently blocked using 1% bovine serum albumin and 0.1% Triton X-100 for 1 h. The primary antibody rabbit anti-NeuN (1 : 500) (Antibody Diluent with Background Reducing Components; Dako Cytomation, Carpenteria, CA, US) were incubated with the sections at 4° C overnight, followed by incubation with the secondary antibody of AlexaFluor 555-labelled goat anti-rabbit antibody to immunoglobulin G (IgG) (1 : 500) at room temperature for 1 h. Subsequently, the sections were counterstained using the nuclear marker 4',6-diamidino-2-phenylindole (DAPI) and placed on slides pretreated with gelatin for subsequent observation. Five randomly selected visual fields in each slice of the cortex and hippocampal CA1 region were photographed for documentation using a fluorescence microscope (Olympus IX71; Olympus Optical, Tokyo, Japan). The number of NeuN-positive cells was counted using the Image J software.

### RNA isolation and quantitation

The total RNA content was extracted using a RNeasy Mini Kit (Qiagen, Valencia, CA, USA). Next, for mRNA detection, the total RNA content was reversely-transcribed into cDNA according to the provided instructions of the Reverse Transcription Kit (RR047A, Takara Bio Inc., Otsu, Shiga, Japan). According to the provided instructions of SYBR^®^ Premix Ex TaqTM II (Perfect Real Time) kit (DRR081, Takara), real time qPCR was conducted in the ABI7500 qPCR instrument (7500, ABI Company, Oyster Bay, NY, USA). The general negative primers of miRNAs and the upstream primers of U6 internal reference were provided by the miRNA First Strand cDNA Synthesis (Tailing Reaction) kit (B532451-0020, Shanghai Sangon Biotechnology Co., Ltd., Shanghai, China), and the other primers were provided by the Shanghai Sangon Biotechnology as shown in Table 2. U6 and glyceraldehyde-3-phosphate dehydrogenase (GAPDH) were regarded as the internal reference for miR-133b and the other genes, respectively. The relative expression of the target gene between the experimental and control groups was calculated based on the 2^-ΔΔCt^ method.

**Table 2:** The primer sequences for RT-qPCR.

**Target**	**Primer Sequences (5'-3')**
miR-133b	F: 5'-GGGTTTGGTCCCCTTCA-3'
	R: 5'-CAGTGCGTGTCGTGGAGT-3'
JAK1	F: 5'-CGACTGACTGCTGGCAACTC-3'
	R: 5'-GACTTGCACTGGCATACTCG-3'
GAPDH	F: 5'- CAAGGTCATCCATGACAACTTTG-3'
	R: 5'- CAAGGTCATCCATGACAACTTTG-3'
Nestin	F: 5'-CTCCTTAGCCACAACCCTCA-3'
	R: 5'-TTCGCAGATTTGGCCCTCAT-3'
MAP-2	F: 5'-TCACTGCTGAGAAAGAGGCA-3'
	R: 5'-ACCTCTTCTGACTCCTTGGC-3'
Tuj-1	F: 5'-GTCCTGGATGTCGTGAGGAA-3'
	R: 5'-CAGTGAGTGAGTGAGCTGGA-3'

Notes: RT-qPCR, reverse transcription quantitative polymerase chain reaction; miR-133b, microRNA-133b; JAK1, Janus kinase-1; GAPDH, glyceraldehyde-3-phosphate dehydrogenase; MAP-2, microtubule-associated protein 2; F, forward; R, reverse.

### Western blot analysis

The total protein content was extracted using the Radio Immunoprecipitation Assay lysis buffer (BB-3209, Shanghai BestBio, Shanghai, China). The proteins were quantified using the bicinchoninic acid (BCA) assay. Subsequently, the 20 μg of protein was subjected to sodium dodecyl sulfate-polyacrylamide gel electrophoresis, and transferred onto the polyvinylidene fluoride membrane at a constant voltage of 80 V. After a blockade for 1 h, the membrane was probed with the primary rabbit polyclonal antibodies to JAK1 (ab133666, 1 : 1000), p-AKT (ab38449, 1 : 500), AKT (ab179463, 1 : 10000), p-GSK-3β (ab75745, 1 : 500), GSK-3β (ab93926, 1 : 500), and WNT-3 (ab172612, 1 : 10000) at 37° C for 1 h. The membrane was then rinsed using PBS 3 times (5 min per time) and re-probed with the goat anti-rabbit horseradish peroxidase (HRP)-labeled immunoglobulin G (IgG) antibody (ab97051, 1: 200) at 37° C for 1 h. The aforementioned antibodies were purchased from Abcam Inc. (Cambridge, UK). Afterwards, the membrane was immersed in the enhanced chemiluminescence (ECL) reaction solution (Pierce Company, Thermo Fisher Scientific, Waltham, MA, USA) at room temperature for 1 min. After removal of the liquid, the membrane was covered with plastic wrap and exposed in dark conditions, and observed after fixation. The relative protein expression was expressed as the ratio of the gray value of the target protein band to the internal reference band (GAPDH served as the internal reference).

### Dual-luciferase reporter gene assay

The dual-luciferase reporter gene assay was conducted to ascertain whether JAK1 was a direct target gene of miR-133b. The dual luciferase reporter gene reporter plasmids of JAK1, namely PGLO-JAK1 WT and PGLO-JAK1 MUT were constructed, both of which were then co-transfected into the HEK293T cells with miR-133b mimic and NC, respectively. After a regime of 24-h transfection, the cells were lysed, and centrifuged at 12000 rpm/min for 1 min to collect the supernatant. The luciferase activity was detected using the Dual-Luciferase^®^ Reporter Assay System (E1910, Promega Corporation, Madison, WI, USA). Each sample was mixed with 100 μL firefly luciferase working solution, and then supplemented with 100 μL of the renilla luciferase detection working solution to detect the relative light unit (RLU). The relative activity of the luciferase was calculated as the ratio of the RLU value (firefly luciferase) to the RLU value (renilla luciferase).

### Statistical analysis

Statistical analysis was performed with the SPSS 21.0 statistical software (IBM Corp. Armonk, NY, USA). All measurement data were expressed as mean ± standard deviation. Comparisons of data between two groups were conducted using the unpaired *t* test. Data among multiple groups were analyzed by means of one-way analysis of variance (ANOVA) with the Tukey’s post hoc test. In all experimental statistics, a value of *p* < 0.05 was considered to be of statistical significance.
